# Medical Opinion and Movement

**Published:** 1906-06-09

**Authors:** 


### Medical Opinion and Movement.

General Miles, who was in command in the Spanish-American war, declared this week at Kansas City that the lives of 3,000 American soldiers were sacrificed in that country from the impurities of the canned food with which they were supplied. He describes the producers of these foods as guilty of a callous fraud, and it is notorious that serious and widespread epidemics of illness are attributable to the use of food which has become infected with organisms capable of producing disease. Drs. Thresh and Porter have dealt with this question exhaustively in a book entitled "Preservatives in Food and Food Examination," which will shortly be published by Messrs. J. and A. Churchill.

We are glad to notice that the London Diocesan Conference has declared that the attitude of Christian Science is antagonistic both to Christianity and Science. Christian Science is condemned from the circumstance that its exponents undertake the treatment of disease without any previous training or knowledge. Their practice is to assure patients at the end of a short interview that pain is non-existent in fact, but a Christian Science quack, when challenged, refused to permit the patient to insert a grain of cayenne pepper into his eye, and has exhibited evidence of sharp suffering when a long pin has been inserted into his person by one of his patient's friends. A Christian Science quack is no less an impostor than the quacksalvers of whom we gave a scandalous instance in last week's issue of The Hospital. It is time that Parliament enacted a law which would lay every impostor of the kind promptly by the heels.

The question "What constitutes professional secrecy" has often been raised in the law courts. A recent case of the kind in Berlin is full of interest and instruction. An unmarried woman, residing with her sister-in-law with a family of children, contracted syphilis. The practitioner in attendance, knowing that the children were in close contact with the infected woman, told the sister-in-law and warned her to keep the children away. Thereupon the infected patient brought an action against the practitioner for violation of professional secrecy, and succeeded in the court below. The supreme court of appeal has, however, reversed this decision on the ground that the practitioner's action in the circumstances was warrantable. It is very desirable that unwarrantable breaches of professional secrecy should be severely punished. But the decision of the Supreme Court of Germany, by defining when disclosure is warrantable, has upheld the true principle of professional conduct, and is likely to prove helpful to the profession throughout the world.

Sir Samuel Wilks, amongst his intimate friends, has long been recognised as the humorist of the medical profession. His geniality, his directness, and his charming personality combine to make him one of the most delightful of companions. Attention has once more been called to the old age excitement caused by Professor Osler's famous utterance by the republication from the *Lancet* of a paper on the subject by Sir Samuel Wilks. Sir Samuel Wilks is intensely humorous in much that he writes, but there is a sober common sense underlying the whole of his theme, which makes it valuable as a contribution to a much debated question. Abernethy held that a man is as old as his arteries, a fact which must modify much which is popularly held to be true of the aged. The members of some families are old men and women before they are 50 years of age, whilst others are young, active, and at their best, intellectually and physically, at 60, or even later. Temperament, the habits of a lifetime, the point of view of the individual, are all material factors which may make for longevity, and do much to maintain physical and bodily health. Each man and woman should learn to know themselves sufficiently to enable them to decide when to retire from active work, for otherwise they may suffer in reputation and become burdensome to their fellows. Each stage of human existence should have its own duties and joys, its opportunities, and occupations. Those who are wise, being of mature age, will ponder these things.

The message of President Roosevelt accompanying the report of the Government Commissioners on the state of affairs in the slaughter and packing-houses at Chicago imposes a duty upon every member of the medical profession throughout the world. It appears from these documents, as the President states, that the present inspection, so far as it has gone, shows the methods adopted in the preparation of food in Chicago to be revolting. They are indeed revolting, for it appears that meat and food products served up in cans and by other methods for the people's consumption are handled with no regard for cleanliness or decency. The details are too horrible to be read without a feeling of nausea, and who would willingly partake of any American or other food with the knowledge that the meat is shovelled from the wooden floors which are filthy beyond description, chopped up on tables which are rarely washed, conveyed in rotten box carts, and mixed with dirt, splinters, floor filth, dead rats, and the expectoration of tuberculous and other diseased workers. These statements are made on the authority of the Government Commissioners appointed by the President, and they should suffice to compel every practitioner to warn his patients and the public generally on every occasion not to permit any American tinned goods or food products to be used in any form in his household so long as the present horrors are permitted to prevail. It has been said that greed has ever been the greatest curse of mankind. The atrocious wickedness of the wealthy people, who have made millions in Chicago by their callous disregard of the claims of their workpeople upon their consideration, and of the duty which they owe to the innocent multitudes who have partaken of these foul foods in good faith, must appal the world. Where conscience is dead and greed takes its place anything is possible, as the official revelations of the actual condition of the Chicago packing-houses prove beyond dispute.

